# Highlight of Immune Pathogenic Response and Hematopathologic Effect in SARS-CoV, MERS-CoV, and SARS-Cov-2 Infection

**DOI:** 10.3389/fimmu.2020.01022

**Published:** 2020-05-12

**Authors:** Yanwen Liang, Mong-Lien Wang, Chian-Shiu Chien, Aliaksandr A. Yarmishyn, Yi-Ping Yang, Wei-Yi Lai, Yung-Hung Luo, Yi-Tsung Lin, Yann-Jang Chen, Pei-Ching Chang, Shih-Hwa Chiou

**Affiliations:** ^1^Department of Medical Research, Taipei Veterans General Hospital, Taipei, Taiwan; ^2^Department of Life Sciences and Institute of Genomic Sciences, National Yang-Ming University, Taipei, Taiwan; ^3^Institute of Food Safety and Health Risk Assessment, National Yang-Ming University, Taipei, Taiwan; ^4^School of Medicine, National Yang-Ming Medical University, Taipei, Taiwan; ^5^Institute of Pharmacology, National Yang-Ming University, Taipei, Taiwan; ^6^School of Pharmaceutical Sciences, National Yang-Ming University, Taipei, Taiwan; ^7^Department of Chest Medicine, Taipei Veterans General Hospital, Taipei, Taiwan; ^8^Division of Infectious Diseases, Department of Medicine, Taipei Veterans General Hospital, Taipei, Taiwan; ^9^Institute of Clinical Medicine, National Yang-Ming University, Taipei, Taiwan; ^10^Department of Pediatrics, Renai Branch, Taipei City Hospital, Taipei, Taiwan; ^11^Institute of Microbiology and Immunology, National Yang-Ming University, Taipei, Taiwan; ^12^Genomic Research Center, Academia Sinica, Taipei, Taiwan

**Keywords:** SARS-CoV, MERS-CoV, SARS-CoV-2, hematopathologic effect, immune responses, immune therapy

## Abstract

A sudden outbreak of COVID-19 caused by a novel coronavirus, SARS-CoV-2, in Wuhan, China in December 2019 quickly grew into a global pandemic, putting at risk not only the global healthcare system, but also the world economy. As the disease continues to spread rapidly, the development of prophylactic and therapeutic approaches is urgently required. Although some progress has been made in understanding the viral structure and invasion mechanism of coronaviruses that may cause severe cases of the syndrome, due to the limited understanding of the immune effects caused by SARS-CoV-2, it is difficult for us to prevent patients from developing acute respiratory distress syndrome (ARDS) and pulmonary fibrosis (PF), the major complications of coronavirus infection. Therefore, any potential treatments should focus not only on direct killing of coronaviruses and prevention strategies by vaccine development, but also on keeping in check the acute immune/inflammatory responses, resulting in ARDS and PF. In addition, potential treatments currently under clinical trials focusing on killing coronaviruses or on developing vaccines preventing coronavirus infection largely ignore the host immune response. However, taking care of SARS-CoV-2 infected patients with ARDS and PF is considered to be the major difficulty. Therefore, further understanding of the host immune response to SARS-CoV-2 is extremely important for clinical resolution and saving medication cost. In addition to a breif overview of the structure, infection mechanism, and possible therapeutic approaches, we summarized and compared the hematopathologic effect and immune responses to SARS-CoV, MERS-CoV, and SARS-CoV-2. We also discussed the indirect immune response caused by SARS and direct infection, replication, and destroying of immune cells by MERS-CoV. The molecular mechanisms of SARS-CoV and MERS-CoV infection-induced lymphopenia or cytokine storm may provide some hint toward fight against SARS-CoV-2, the novel coronavirus. This may provide guidance over using immune therapy as a combined treatment to prevent patients developing severe respiratory syndrome and largely reduce complications.

## Introduction

Coronaviruses belong to the *Coronaviridae* family of the subfamily *Coronavirinae*. The viruses of this family have a broad range of animal hosts, and zoonotic transfer between species is common. Within the *Coronavirinae* subfamily, there are four genera: *Alphacoronavirus, Betacoronavirus, Gammacoronavirus*, and *Deltacoronavirus* (1, 2). Coronaviruses are non-segmented positive-sense RNA viruses, whose RNA is covered by the solar corona-shaped envelope, from which they acquired their name. They are characterized by having the largest genome among all RNA viruses with an average size of 30 kb (3). Two-thirds of the coronaviral genome encodes non-structural proteins responsible for the virus replication, including RNA-dependent RNA polymerase, proteases, and helicase. The 3′ end of the genome encodes four main structural proteins of the coronavirus particles, which are the spike (S), membrane (M), envelope (E), and nucleocapsid (N) proteins (4).

Coronaviruses have a long history of infecting humans. HCoV-229E, HCoV-OC43, HCoV-NL63, and HCoV-HKU1 are the prevalent human coronaviruses, which are estimated to have been circulating in the human population for centuries (4). These viruses cause mild upper respiratory infection, or in other words, common cold symptoms (5). On the other hand, three members of the *Betacoronavirus* genus were zoonotically transferred to humans from other mammalian species in the past two decades and caused major epidemics with high mortality rates. Severe Acute Respiratory Syndrome (SARS), caused by SARS-CoV, started in Guangdong province of China in 2002 and affected 8,096 people worldwide, resulting in 774 deaths (10% mortality rate) (https://www.cdc.gov/sars/about/faq.html). Middle East respiratory syndrome (MERS) caused by MERS-CoV started in Saudi Arabia in 2012 and affected 2,506 people, causing 862 deaths worldwide with a 35% mortality rate (https://www.who.int/csr/don/31-january-2020-mers-united-arab-emirates/en/). In December 2019, a novel coronavirus, SARS-CoV-2, caused an outbreak of Coronavirus Disease 2019 (COVID-19) in Wuhan city in China, which quickly spread throughout the world and grew into a global pandemic affecting hundreds of thousands of people as of March 2020. Notably, although SARS-CoV-2 is characterized by higher contagiousness in comparison with SARS-CoV and MERS-CoV, it causes a much lower mortality rate (2.3% from the epidemic in China in Jan.-Feb, 2020) (6). All three viruses can cause acute respiratory distress syndrome (ARDS), the most acute and fatal stage of the disease, characterized by wide-spread inflammation in the lungs resulting from the aberrant immune response to the viral infection (7–9).

Therefore, in this review, we discuss three coronaviruses, SARS-CoV, MERS-CoV, and SARS CoV-2, from an immunological point of view. We describe their structure and protein composition, mechanisms of entering host cells, and mechanisms to evade innate immune responses. Comparing their hosts, invading mechanisms, and inflammatory responses will help us understand more about coronaviruses, aid in solving the global SARS-CoV-2 epidemic happening now, and find out possible effective treatments to deal with the public health crises caused by coronaviruses in the future.

## Virus Structure

As was demonstrated by cryoelectron tomography and cryoelectron microscopy, coronavirus virions are of spherical shape with diameters of approximately 65–125 nm (10). The club-shaped spikes on the surface of the virion are the most prominent feature of coronaviruses. These spikes confer them a solar corona-like appearance from which the name “coronavirus” is derived. The nucleocapsids are helically symmetrical and are packed by the envelope of the virion (5). Coronavirus particles contain four main structural proteins, namely the spike (S), membrane (M), envelope (E), and nucleocapsid (N) proteins.

## S Protein

Coronavirus S protein is a large multifunctional class I viral transmembrane protein, whose size varies from 1,160 amino acids in Infectious Bronchitis Virus (IBV) in poultry to 1,400 amino acids in Feline Coronavirus (FCoV) (11). It is a trimer located on the virion surface, giving the virion a crown-like appearance. As for its function, it mediates the entry of the infectious virion particles into the cells by making attachments between virion particles and host cell membranes through interaction with various host cellular receptors (12). Furthermore, it plays an important role in tissue tropism and the determination of host range (13). In addition, S protein is capable of inducing host immune response (13). S proteins in all coronaviruses can be divided into two domains, S1 and S2 (11). S1 functions as the receptor-binding domain (RBD) while S2 acts as a membrane fusion subunit. The S1 domain can be further divided into two subdomains, named the N-terminal domain (NTD) and the C-terminal domain (CTD). Both of these subdomains act as the receptor-binding domains, interacting efficiently with various host receptors (13). The S1 CTD contains the receptor-binding motif (RBM).

## M Protein

The M protein is the most abundant structural protein of the coronavirus virion. It is a small (~25–30 kDa) protein with three transmembrane domains that is responsible for maintaining the shape of the virion (14). The amino acid sequences of the M protein are diverse in different coronaviruses, however, the structural similarity is maintained overall (15). It has a short N-terminal glycosylated domain outside the virion and a much larger C-terminal domain inside the virion that extends 6–8 nm into the viral particle (16). Most M proteins are co-translationally inserted into the ER membrane without a signal sequence. The viral scaffold is maintained by interactions between M proteins. Recent studies suggest that the M protein exists as a dimer in the virion, and may adopt two different conformations allowing it to promote membrane curvature, as well as bind to the nucleocapsid (14).

## E Protein

The E protein is the smallest structural protein (~8–12 kDa) within the virion. It plays a multifunctional role in the pathogenesis, assembly, and release of the virus. The virulence of the virus is also related to the E protein (17). The E proteins from different coronaviruses are highly diverse in their amino acid sequences but are characterized by a common structure (18). There are three domains in the E protein: short hydrophilic amino-terminal domain, large hydrophobic transmembrane domain, and C terminal domain (19). The deletion of the E protein-encoding gene results in slower amplification of the virus, but the protein does not seem to be essential for the replication of SARS-CoV (20). Besides its role in assembly and release of the virus, the E protein still has other functions, for instance, the ion channel activity. Compared to SARS-CoV, the SARS-CoV-2 (2019-nCoV) E protein reveals a similar amino acid constitution without any substitution (21).

## N Protein

The N protein is the only structural protein present in the nucleocapsid. It is composed of three highly conserved and separate domains: an N-terminal domain (NTD), RNA-binding domain or a linker region (LKR), and a C-terminal domain (CTD) (22). The NTD binds to the 3′ end of the viral RNA and is highly divergent from virus to virus (23). The LKR region [also called SR (Serine and Arginine) domain] is charged because of its serine and arginine-rich sequence (24). It has been reported to interact directly with RNA *in vitro* and play a part in cell signaling (25, 26). The N protein has two RNA substrates that have already been identified, the transcriptional regulatory sequence (TRS) (25) and the genomic packaging signal (27). In addition, it can also act as a viral suppressor of RNA silencing in mammalian cells (28). N protein is also heavily phosphorylated (29), so that it can change its conformation to enhance the affinity for viral vs. non-viral RNA. N protein also binds nsp3 (24, 30) and the M protein (31). These proteins may interact to help tether the viral genome packaging.

## HE Protein

The hemagglutinin-esterase (HE) is a structural protein present in a subset of *Betacoronavirus*. The protein acts as a hemagglutinin, which binds sialic acids of surface glycoproteins. It also contains acetylesterase activity (32). These activities are thought to enhance the cell entry mediated by the S protein and virus spread through the mucosa (33).

## Structure OF SARS-CoV

SARS-CoV virus particles are spherical with an average diameter of 78 nm. The virus contains a helical nucleocapsid, surrounded by an envelope (34), covered with rod-shaped long envelope particles of about 20 nm in length, with typical coronal features. The structure of SARS-CoV is similar to that of other coronaviruses. The gene sequence is 5′ end, replicase [rep], spike [S], envelope [E], membrane [M], nucleocapsid [N], 3′ end. There are short untranslated regions at both ends. The sequences of the other five non-structural proteins may be distributed between ORF S and N (35).

The SARS-CoV genome contains a total of 11 ORFs and encodes 23 mature proteins (36). Among them, two major ORFs (ORF1a and ORF1b) account for about two-thirds of the genome size and encode two important polyproteins, pp1a and pp1ab. Polyproteins are proteolytically cleaved to produce non-structural proteins, the most important of which are RNA-dependent RNA polymerase and ATPase helicase. Only several nucleotides are different among different viruses (37).

## Structure OF MERS-CoV

The genome of MERS-CoV consists of genes encoding the replicase and structural proteins (spike-envelope-membrane-nucleocapsid)-poly (A)−3′, similar to other coronaviruses. The virus has 10 ORFs and encodes 16 putative non-structural proteins involved in the viral transcription and replication process (38, 39).

## Structure OF SARS-CoV-2

Basically, the structure of SARS-CoV-2 shares all the typical characteristics with other coronaviruses. Several recent studies considering the structure of SARS-CoV-2 were all focused on the S protein. Wrapp et al. (40) reported a structure at 3.5 Å resolution of SARS-CoV-2 S protein. Yan et al. (41) reported the complex structure of B^0^AT1, an amino acid transporter protein, with human host cell binding receptor angiotensin-converting enzyme 2 (ACE2), which provided important insights into the molecular basis of coronavirus infection. Lan et al. (42) reported a crystal structure of SARS-CoV-2 S protein's receptor binding domain (RBD) region bound to ACE2. The viral architecture of SARS-CoV-2 with post-fusion spike was observed by Cyro-EM, which showed the image of disassociated spikes (43).

## Infection (Entering Host Cells)

### SARS-CoV

Similar to other coronaviruses, SARS-CoV enters cells through endocytosis and membrane fusion, and its host receptor is ACE2 (35, 44). SARS-CoV enters into target cells and can be inhibited by polyanionic compounds, suggesting that the SARS-CoV envelope protein may be positively charged. At the same time, SARS-CoV needs to be in the acidified endosome to produce effective infection, indicating that its effect is pH-dependent (45). Viral RNA is replicated in the unique bottle-shaped bilayer membrane compartments (46). Several studies have found that SARS-CoV infection can cause ultrastructural changes *in vivo* and in cultured cells, including the formation of double-membrane vesicles and nucleocapsid inclusions and particles in the cytoplasm (34).

### MERS-CoV

MERS-CoV has been reported as being able to infect and kill not only alveolar epithelial cells but also T cells (47). MERS-CoV enters host cells by binding to a DPP4 receptor expressed in the kidney and other organs (48), and uses proteases of the host to enter lung cells. Furin activates the S protein on the viral envelope, mediating the membrane fusion and virus entry into host cells (49). Like SARS-CoV, MERS-CoV can overcome the host's natural immune response, produce high virus titers, and induce cytokine imbalance (38, 50).

### SARS-CoV-2

**Figure 1:**
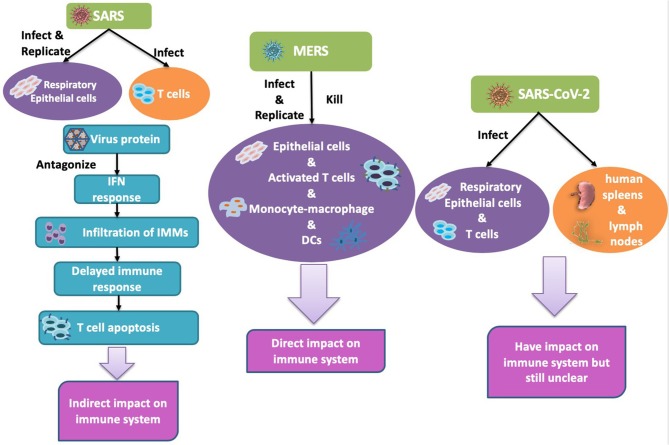
Human coronavirus infects different types of cells. **Left**: SARS-CoV can infect alveolar epithelial cells and immune cells but can only replicate in epithelial cells. **Middle**: MERS-CoV infected and replicated in both alveolar epithelial cells and immune cells. **Right**: SARS-CoV2: infected lung and damaged lung and immune system.

SARS-CoV-2 is mainly considered to infect respiratory epithelial cells, but a recent study confirmed that it can also infect T lymphocytes (51), spleens, and lymph nodes (52). There are already some solid studies that confirm that ACE2 serves as the receptor for the entry of SARS-CoV-2. Analysis of the receptor binding motif (RBM), a portion of the receptor binding domain (RBD) that makes contact with ACE2 (53), revealed that most amino acid residues essential for ACE2 binding by SARS-CoV were conserved in SARS-CoV-2. Hoffmann et al. blocked ACE2 in Vero cells and found that both SARS-CoV and SARS-CoV-2 infection was dramatically inhibited, and that serine protease TMPRSS2 played an important role in SARS-CoV-2's infection (54). The difference of the host cells among SARSCoV, MERS-CoV and SARS-CoV-2 is summarized in Figure 1.

### ACE2 Receptor

Angiotensin-converting enzyme 2 (ACE2) is an essential component of the renin-angiotensin system (55). It was shown to bind to the S protein of SARS-CoV in 2003 by mass spectrometry (56) and was also confirmed to be a receptor of SARS-CoV-2 required to enter human cells (57). Xu et al. (58) drafted the currently available world's largest human kidney cell atlas with 42,589 cells and identified 19 clusters through unsupervised hierarchical clustering analysis. ACE2 and TMPRSS genes were significantly co-expressed in podocytes and proximal convoluted tubules as potential host cells targeted by SARS-CoV-2. Comparative analysis showed that ACE2 expression in kidney cells was no less than that in the lung, esophagus, small intestine, and colon, suggesting that the kidney may be an important target organ for SARS-CoV-2.

As for the susceptibility of different population groups to SARS-CoV-2, Chen et al. (59) showed that the expression of ACE2 in Asians was similar to that in other races, and was also not related to sex. Surprisingly, ACE2 was shown to be significantly upregulated after virus infection, including SARS-CoV and SARS-CoV-2 (60). According to the public data analysis, the level of ACE2 expression in adipose tissue was higher than that in lung tissue, which was indicative of the possibility that adipose tissue was also a potential target of SARS-CoV-2 (61).

### Inflammatory Response to Coronavisuses

Human coronaviruses can be divided into two groups by their pathogenicity. Whereas, low pathogenic coronaviruses (HCoV-OC43, HCoV-229E, HCoV-NL63, and HCoV-HKU1) cause mild cold-like respiratory illness, the highly pathogenic SARS-CoV, MERS-CoV and SARS-CoV-2 cause immunopathological events that result in fatal pneumonia. The invasion of such coronaviruses is associated with severe immune responses, which may eventually lead to acute respiratory distress syndrome (ARDS). The innate immune system constitutes the primary line of defense against the invading viruses. The pathogen-associated molecular patterns (PAMPs), represented by the viral RNA or dsRNA formed during viral replication, are recognized by intracellular sensors such as RIG-I and MDA5. After recognition, the downstream signaling cascade results in activation of NF-κB and IRF3 transcriptional activity (62). This leads to the expression of type I interferon (IFN) and pro-inflammatory cytokines, which constitute the defense line against the virus infection at an early stage (63).

SARS-CoV and MERS-CoV have evolved a number of strategies to suppress type I IFN response during their invasion. SARS-CoV can interfere with the downstream signaling of the RNA sensors, including MAVS and TRAF3/6, directly or indirectly (64). As for MERS-CoV, it can downregulate interferon-stimulated genes (ISG) by activating repressive histone modification as its strategy (64).

As part of the adaptive immunity, T cells also play important roles in the primary defense line against coronaviruses. There are many T cell epitopes identified to induce an IFN-γ-specific T cell response or cytotoxic T lymphocyte (CTL) response. Studies have found that epitopes in the S protein (64, 65) and the N protein of coronaviruses (66, 67) can induce antibody responses in both mice models or patients. IgM and IgG, produced by B lymphocytes, are formed after the infection of coronaviruses (68, 69). The induction of IgM is an early and transient response to neoantigens, which is later replaced by the induction of IgG to play the role as the predominant and long-term antibody. IgG is characterized by a longer half-life and lower molecular weight, which gives it the ability to provide long-lasting protection and effective tissue penetration (68).

### The Immune Response to SARS-CoV

The combined induction of antibodies and virus-specific T cells provides optimal protective immunity. Following the infection, a strong humoral immune response with a high titer of neutralization antibodies targeting the SARS-CoV S protein that show a protective effect are found in the serum of most patients. In addition, CD4+ T cells targeting N protein and HLA-A2 restricted CD8+ T cells targeting S protein were observed in SARS patients (70–72). However, the dramatic loss of CD4+ T cells (in ~90–100% of patients) and CD8+ T cells (in ~80–90% of patients) was observed in the acute phase of SARS patients (73). The delayed adaptive immune response resulted in prolonged virus clearance and correlated with the severity of the SARS disease (74). One possible reason for the decreased number of T cells is that after infecting alveolar epithelial cells, SARS-CoV encodes multiple structural and non-structural proteins that antagonize innate IFN response (75–78). The delayed IFN response orchestrates infiltration of pathogenic inflammatory monocyte-macrophages (IMMs) and elevation of pro-inflammatory cytokines (79). IMM-derived pro-inflammatory cytokines, such as type I INF (80), may sensitize T cells to undergo apoptosis through Bim (81) or Bcl-xL (82)-mediated intrinsic pathway by E protein, thus consequently impeding the viral clearance (79). Depletion of IMMs or neutralization of pro-inflammatory cytokines was shown to protect mice from lethal SARS-CoV infection (79). Another possible explanation of the reduction of virus-specific T cells is the alteration in antigen presenting cell (APC) function and impaired dendritic cell (DC) migration, resulting in the reduced priming of T Cells (83, 84). This mechanism was supported by animal studies using SARS-CoV-MA15, the mouse-adapted strain of SARS-CoV. Inefficient activation of respiratory DCs by SARS-CoV-MA15 attributed to poor virus-specific CD4+ and CD8+ T cells responses (84). Moreover, the age-dependent reduction in the magnitude of T cell response may also explain the higher susceptibility to SARS-CoV with advanced age (85). Consistently, depletion of CD4+ T cells delayed SARS-CoV (Urbani strain) clearance and enhanced pneumonitis (86). In contrast, transfer of SARS-CoV-specific CD4+ and CD8+ T cells resulted in rapid virus clearance and amelioration of the disease (87). Mechanistically, the pattern recognition receptors such as MyD88 (88) and TRIF (89) are required for protection against SARS-CoV infection.

In addition to the humoral response, a 3-year follow-up study of 176 SARS patients showed that the level of IgM peaked at ~1 month after symptoms onset, and IgG peaked at 2–4 months (90). Patients with a longer illness period showed a lower neutralizing antibody response compared to patients with a shorter illness duration (91). It was reported that vaccine-elicited, neutralizing monoclonal antibody (MAb) targeting the S protein of SARS-CoV facilitates viral entry into host cells and enhances viral infectivity (92). This phenomenon is the so called antibody-dependent enhancement (ADE) (69), which is regarded as a great burden for vaccine development.

### The Immune Response to MERS-CoV

The immune response mechanism triggered by MERS-CoV has still not been fully studied. It is known that the S protein of MERS-CoV can upregulate the levels of the repressors of the TLR signaling pathways, such as of IL-1R-associated kinase (IRAK-M) and peroxisome proliferator-activated receptor-gamma (PPARY). IRAK-M and PPARY negatively regulate IRF7, which normally induces the expression of IFN-alpha and IFN-beta (93). If these negative regulators can maintain their persistence in the long-term, the clearance of MERS-CoV infections will be impaired.

Comparatively less is known about the fate of T cells in MERS-CoV infection and little information is known about the recognized epitopes (72). Similar to SARS-CoV, MERS-CoV-specific CD8+ T cells are also important for clearing the virus (94). Though both SARS-CoV and MERS-CoV infects monocyte-macrophages, DCs, and activated T cells, only MERS-CoV was able to replicate in the infected immune cells, which consequently resulted in aberrant induction of inflammatory cytokines in macrophages and DCs (95, 96) and of both extrinsic and intrinsic apoptosis pathway in T cells (47). Such active replication of MERS-CoV in these immune cells may underlie the comparatively higher fatality rate of MERS disease.

As for humoral immunity, antibody response to MERS-CoV is typically detected on the second and third week after the onset of infection. But the longevity of the antibodies seemed to be correlated to the severity of disease. In patients who had pneumonia caused by MERS-CoV, the antibodies were still detectable 13 months after infection (97). However, in patients after mild or subclinical infection of MERS-CoV, MERS antibodies were detected at low levels (98). Similar to SARS-CoV, MAb that has a strong binding affinity to the spike protein of MERS-CoV also facilitates ADE viral entry into host cells (99).

### The Immune Response to SARS-CoV-2

According to case reports, the pathogenesis of SARS-CoV-2 includes immunological responses of both innate and adaptive immunity systems.

Compared to normal patients, patients requiring ICU admission had higher concentrations of GCSF, IP10, MCP1, MIP1A, and TNFα. These cytokines may help to judge the condition of patients (100).

Secondary hemophagocytic lymphohistiocytosis (sHLH), which is mostly triggered by virus infection in adults, is a condition in which the body makes too many activated immune cells (macrophages and lymphocytes) (https://primaryimmune.org/disease/hemophagocytic-lymphohistiocytosis-hlh). The cytokine profile of sHLH is associated with the severity of COVID-19, which is characterized by increased interleukin (IL)-2, IL-7, GCSF, IP10 (CXCL10), MCF1 (CCL2), MIP1A (CCL3), and TNF-α (100, 101). Therefore, it is possible that this phenomenon happens in COVID-19 patients. The current explanation for the sHLH phenomenon is that the body has experienced a cytokine storm caused by excessive immunity. However, the details of immune and inflammatory response to SARS-CoV-2 infection are still under scrutiny.

Similar to SARS-CoV, SARS-CoV-2 also targeted pneumocytes (both types I and II) and alveolar macrophages (102). Consistently, pathological examination of patients who were infected by SARS-CoV-2 revealed the infiltration of plasma cells and macrophages and a high density of macrophages and foam cells in the alveolar cavities (103). However, compared with SARS-CoV, SARS-CoV-2 did not significantly induce types I, II, or III interferons in the infected human lung tissues (102). As cytokine storm may be the main cause for the severity of the coronavirus infection, these findings support the relevant severity of SARS-CoV and SARS-CoV-2.

As for adaptive immunity, it is known that the low levels of CD4+ and CD8+ T cells are related to the mortality of SARS-CoV-2 patients (104, 105). The up-regulation of apoptosis and autophagy in PBMC of SARS-CoV-2 patients (106) suggested that, similar to how MERS can directly infect T cells and induce apoptosis (47), SARS-CoV-2 may cause lymphocytopenia through inducing T-cell apoptosis or autophagic cell death. It was supported by a recent report showing that SARS-CoV-2 could infect T cells through receptor-dependent or S protein-mediated membrane fusion (51).

As for the antibody response, it was reported that in 23 patients with COVID-19, the viral load peaked during the first week and then began to fall. Both IgG and IgM antibodies which targeted the nucleoprotein and the surface spike receptor began to rise around 10 days after symptom onset, and the seroconversion of most patients happened within the first 3 weeks (107). Another study among 173 patients reported that the seroconversion rate of Ab, IgM, and IgG was 93.1, 82.7, and 64.7%, respectively. And the median seroconversion time for Ab, IgM, and IgG were day 11, day 12, and day 14 after onset, respectively (108). Whether ADE can happen in SARS-CoV-2 infection is still not confirmed, but as humans have already experienced a SARS-CoV epidemic and several other coronavirus infection such as 229E (109), according to former studies of SARS-CoV (92), it is possible that ADE can also happen in the infection of SARS-CoV-2 (110).

## Discussion and Summary

The recent outbreak of the SARS-CoV-2 infection has caused a worldwide crisis in the epidemiology and medical systems. Since SARS-CoV-2 was confirmed to share the same host receptor, ACE2, with SARS-CoV, the strategies used to tackle SARS-CoV are under investigation for treating SARS-CoV-2 infection. However, despite both attacking lungs and using the same host receptor to enter target cells, the three coronaviruses causing three serious pneumonia epidemics are different in the range of infected cell types and their effects on infected cells.

SARS-CoV is mainly replicated in respiratory epithelial cells, though it can also infect a variety of immune cells such as monocytes, macrophages, dendritic cells, and activated T cells (111–114). MERS-CoV, in contrast, not only infects the immune cells and epithelial cells, but is also able to replicate in the former cells and lyse them, which may be one of the reasons for the high mortality of MERS (47, 96, 115). The details of the infection and lytic replication mechanisms of SARS-CoV-2 in host cells are currently unclear (Table 1). However, diarrhea, liver and kidney damage, and hemophagocytic lymphohistiocytosis have been reported in patients with SARS-CoV-2, indicating that the host cell range of the virus may be wider than currently recognized (100).

**Table 1:** Immunology differences between SARS-CoV, MERS-CoV, and SARS-CoV-2.

	**SARS**	**MERS**	**SARS-CoV-2**
Infected host cell	Alveolar epithelial cells Monocyte-macrophage Dendritic cells Activated T cells	Alveolar epithelial cells Monocyte-macrophage Dendritic cells Activated T cells	Respiratory epithelial cells T lymphocytes
Suspectable for virus replication	Respiratory epithelial cells	Alveolar epithelial cells Monocyte-macrophage Dendritic cells Activated T cells	Respiratory epithelial cells Unknown
Monocyte-macrophage	Abortive replication Viral protein inhibition	Viral replication Kill monocyte-macrophage	Unknown
Impacts on immune system	Indirectly kill: T cell apoptosis	Viral replication Directly killed T cell	Unknown

**Figure 2:**
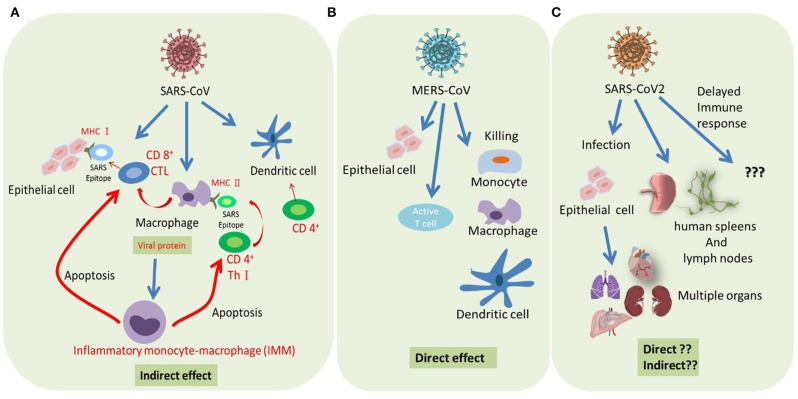
Summary of host immune response modulated by severe coronaviruses. **(A)** SARS-CoV infected epithelial cells represents SARS epitope by MHC I to recruit CD8+ cytotoxic T cells (CTL). Macrophage and dendritic cells (DCs) are infected by SARS-CoV and represent SARS epitope by MHC II to recruit CD4+ helper T cells (Th1). Abortive replication of SARS in macrophage impaired its cytokine production, resulting in a delayed IFN response, infiltration of inflammatory monocyte-macrophages (IMMs), and T cells apoptosis. In addition, SARS-CoV infection impaired dendritic cell (DC) function, resulting in reduced T cell activation. **(B)** Successful replication of MERS-CoV in both alveolar epithelial cells and immune cells resulted in the direct killing of these infected cells. **(C)** SARS-CoV-2 can probably infect both lung epithelial cells and immune cells and damage the tissue through a direct or cytokine-mediated indirect effect.

Following the invasion of a pathogen, the host triggers a serious response from the immune system. SARS-CoV does not directly lyse and kill T cells, but indirectly induces T cell apoptosis (82). MERS-CoV, which is a more severe and aggressive virus, directly targets T cells and undergoes lytic replication, thus directly causing their death (47). Therefore, MERS-CoV has a direct impact on the immune system. SARS-CoV-2 is reported to infect T lymphocytes (51), but we still don't know how this affects the immune system in particular (Figure 2). A severe reduction of immune cells was observed in patients infected with SARS-CoV-2, but whether this phenomenon is directly caused by the virus or indirectly caused by dysregulated cytokine production by immune cells has yet to be determined (100). Direct viral effects require treatment strategies that target viral replication. Indirect viral effects through dysregulated cytokine production by residential macrophages/dendritic cells or antigen presentation by APCs can now be treated by immune therapy approaches. Understanding the behavior of SARS-CoV-2 in the host cells of the human body and its effect on the immune system may provide important tips for combating the disease.

There have already been some studies showing that other tissues and organs may also be the target of SARS-CoV-2, further reminding us to focus on other organs besides lungs, such as the kidneys, spleen, and lymph nodes (52, 116). This may give us some hints for the multiple organ dysfunction syndrome in some severe COVID-19 cases (117). Moreover, research on the expression pattern of ACE2 in different population groups and races indicates that there is no sex or race bias in susceptibility to SARS-CoV-2 (118).

It is still unclear whether reinfection with SARS-CoV-2 can occur in recovered patients. There has been some news about “reoccurring COVID-19 cases” (https://www.scmp.com/news/china/society/article/3065091/coronavirus-recovered-patient-dies-china-reports-139-new-cases), but as they are not formal case reports, it is not certain whether these patients had fully recovered from COVID-19 before the symptoms relapse. In a study on rhesus macaques re-exposed to SARS-CoV-2 after disappearance of symptoms and positive antibody response to primary infection, no evidence of reinfection was found (119). Also, how long the antibodies will remain in recovered patients is still unclear. Since the vaccine against COVID-19 has still not been developed, the recommendations of the CDC, which advises people to wear cloth face masks and keep a 6-foot distance from others, should be the best way to prevent reinfection (https://www.cdc.gov/coronavirus/2019-ncov/prevent-getting-sick/diy-cloth-face-coverings.html).

To deepen the research on coronavirus, humans must learn enough lessons from this pandemic. In the wave of globalization and scientific and technological progress, infectious diseases have become more prone to spreading, which has made it harder for humans to deal with them. How to understand the infectious biomolecular mechanism and immune pathological environment in the future will be a more important proposition for us than ever before. According to China's response to the epidemic, it did not take long to identify what the pathogen was, but the imperfect public health emergency system is the main reason for the spread of the epidemic. Therefore, in addition to developing drugs, vaccines, and updating treatment plans, scholars should also call on governments to strengthen the construction and improvement of social public health emergency systems to prevent similar pandemics from happening again.

## Author Contributions

S-HC and P-CC designed, constructed, and organized this work, and reviewed article. YL, M-LW, C-SC, Y-PY, and AY collected all references and wrote up the manuscript. W-YL, Y-HL, Y-TL and Y-JC prepared the study materials and organized figures and table.

## Conflict of Interest

The authors declare that the research was conducted in the absence of any commercial or financial relationships that could be construed as a potential conflict of interest.
